# Supplementary material for: Balance of Macrophage Activation by a Complex Coacervate-Based Adhesive Drug Carrier Facilitates Diabetic Wound Healing
Source: Antioxidants (Basel). 2022 Nov 28;11(12):2351. doi: 10.3390/antiox11122351 (PMC9774176; doi:10.3390/antiox11122351)
Supplement: Supplementary file 1 [file antioxidants-11-02351-s001.zip › antioxidants-2020778-supplementary.pdf]

Supplemental Results

Title: Balance of macrophage activation by a complex coacervate-based adhesive drug carrier facilitates diabetic wound healing

Authors: Ching-Shuen Wang[1], Shen-Dean Luo[2], Shi-hai Jia[3], Chen-Han Wilfred Wu[4], Shwu-Fen Chang[5], Sheng-Wei Feng[1], Chieh-Hsiang Yang[6], Jiann-Her Lin[7] and Yinshen Wee[8*]

1 School of Dentistry, College of Oral Medicine, Taipei Medical University, Taipei 110, Taiwan.

2 Department of Otolaryngology, Kaohsiung Chang Gung Memorial Hospital and Chang Gung University College of Medicine, Kaohsiung , Taiwan.

3 Department of Neurobiology University of Utah, Salt Lake City, UT 84112, USA.

4 Department of Genetics and Genome Sciences, School of Medicine, Case Western University, Cleveland OH 44106, USA.

5 Graduate Institute of Medical Sciences, College of Medicine, Taipei Medical University, Taipei 110, Taiwan.

6 Department of Oncological Sciences, University of Utah, Salt Lake City, UT 84112, USA.

7 Department of Neurosurgery, Taipei Medical University Hospital, Taipei 110301, Taiwan.

8 Department of Pathology, University of Utah, Salt Lake City, UT 84112, USA.

*Correspondence: yin.wee@hsc.utah.edu; Tel.: +01-801-213-4217.

Keywords: complex coacervates, oligochitosan, phytic acid, sustained release, diabetic wound healing, M2 macrophage polarization, db/db diabetic model.

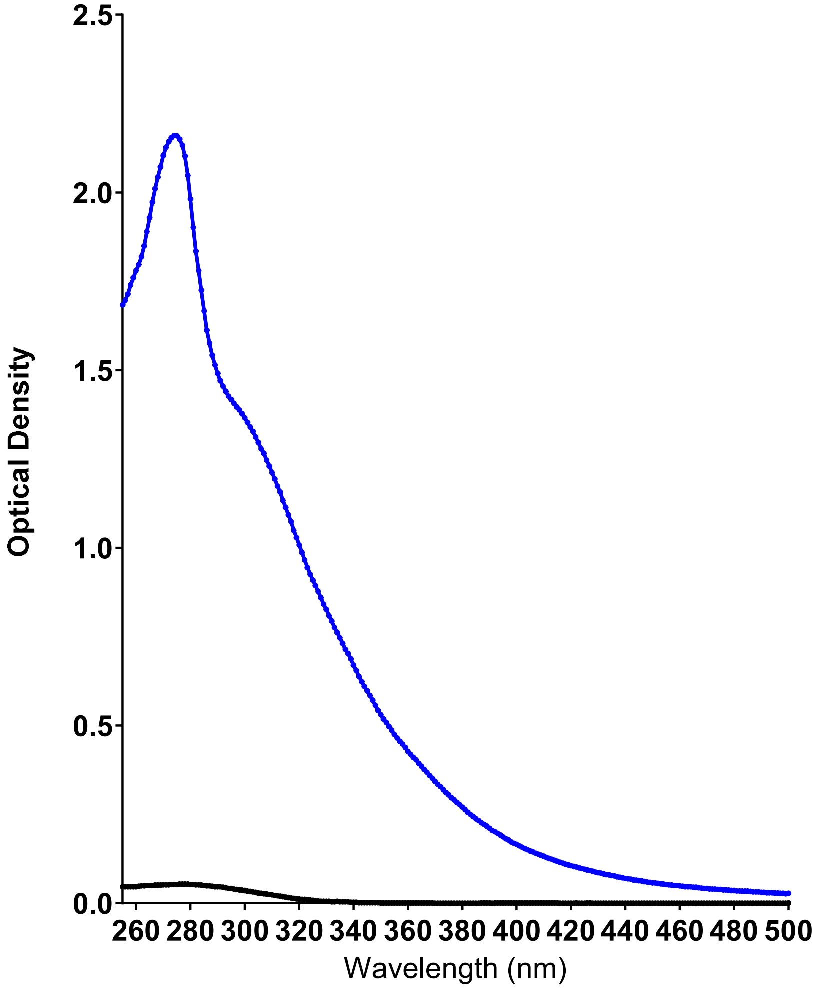

Fig. S1. UV spectrum of Och and IP6. The UV–Vis spectra (240-500nm) of Och (blue line) and IP6 (black line) solution.

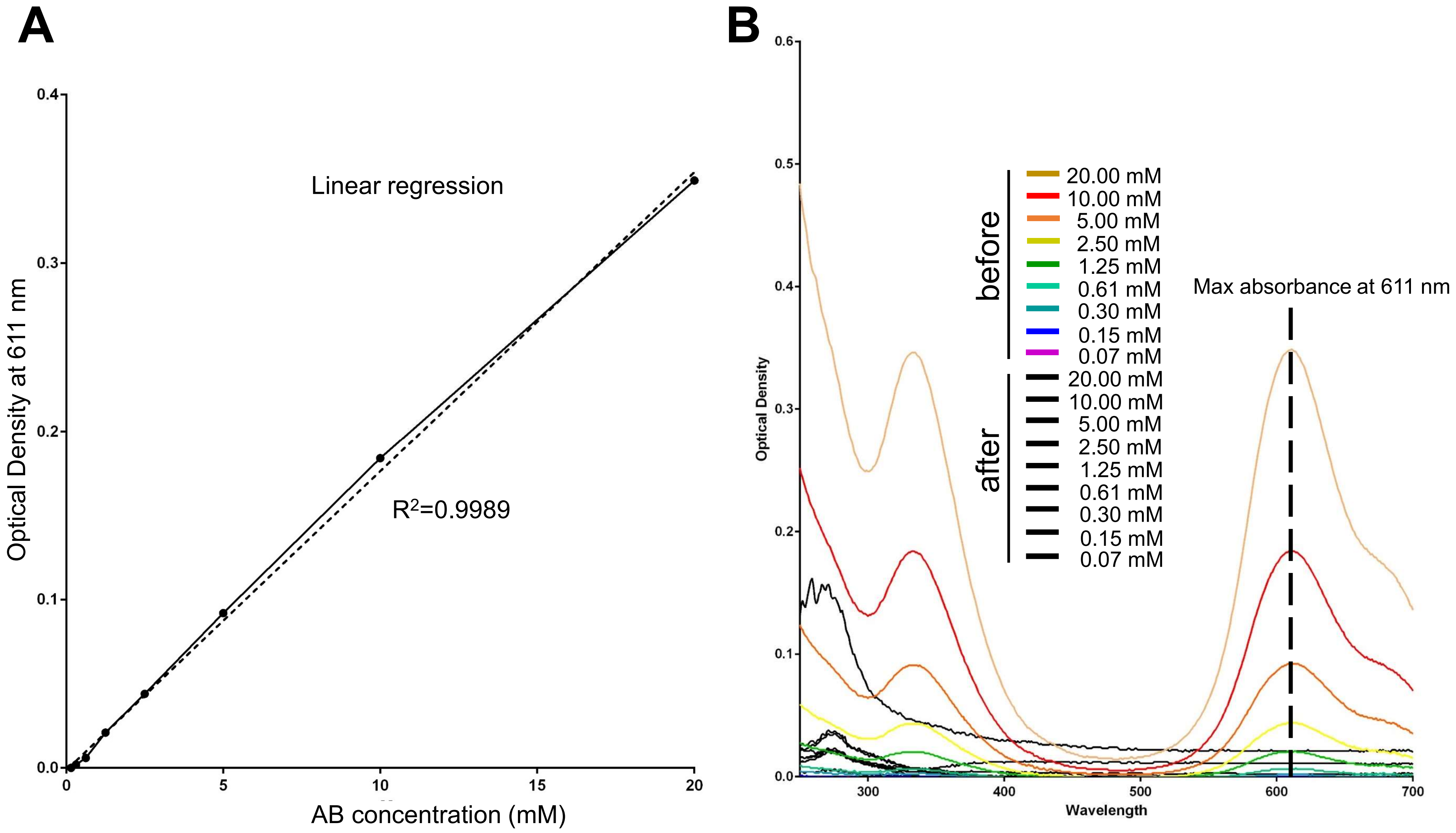

Fig. S2. The encapsulation capacity of Alcian Blue (AB) into the ADC system. (A) UV absorbance intensity as a function of AB concentration in solution. (B) The UV–Vis spectra of AB solution before and after the loading into ADC system.

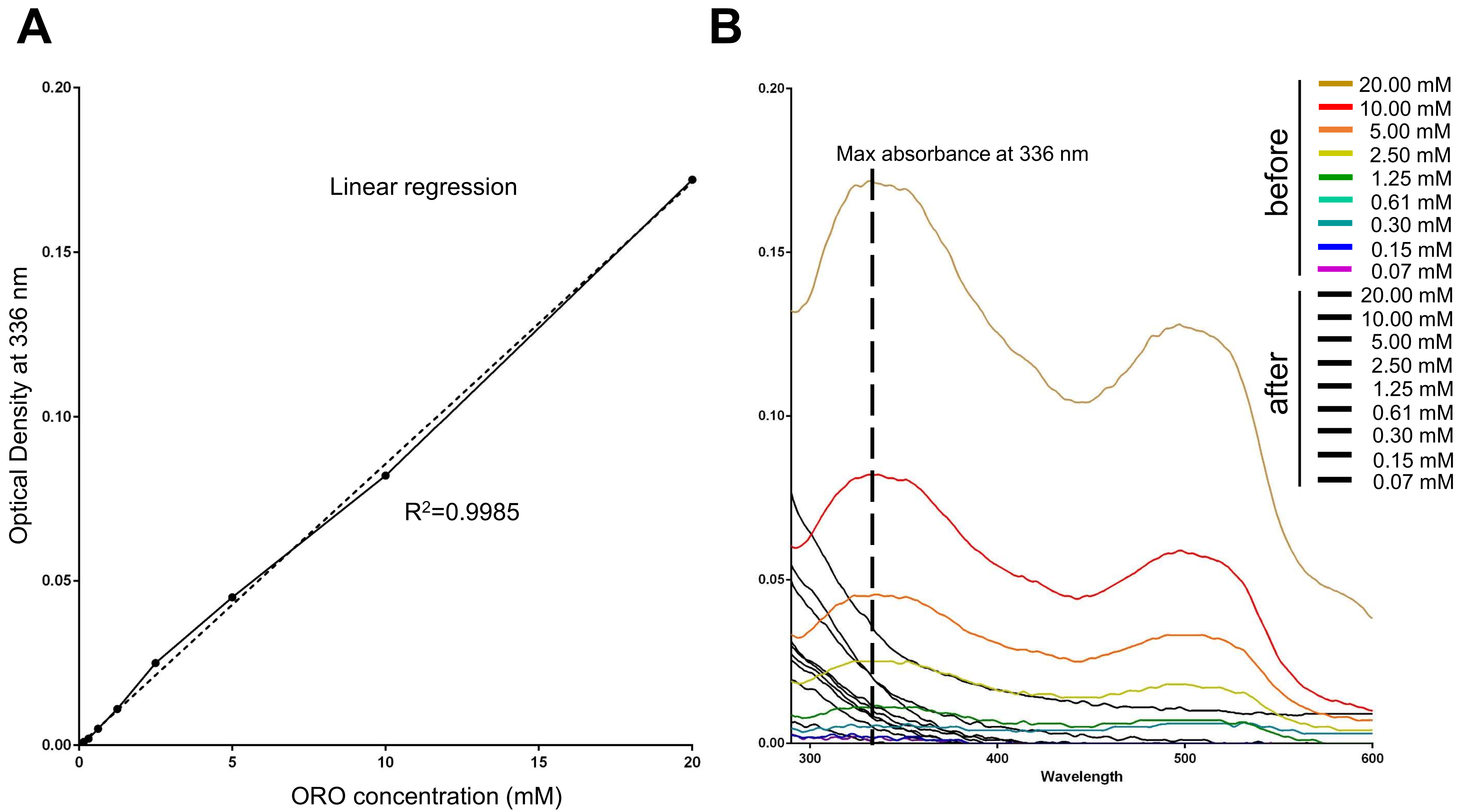

Fig. S3. The encapsulation capacity of Oil Red O (ORO) into the ADC system. (A) UV absorbance intensity as a function of ORO concentration in solution. (B) The UV–Vis spectra of ORO solution before and after the loading into ADC system.

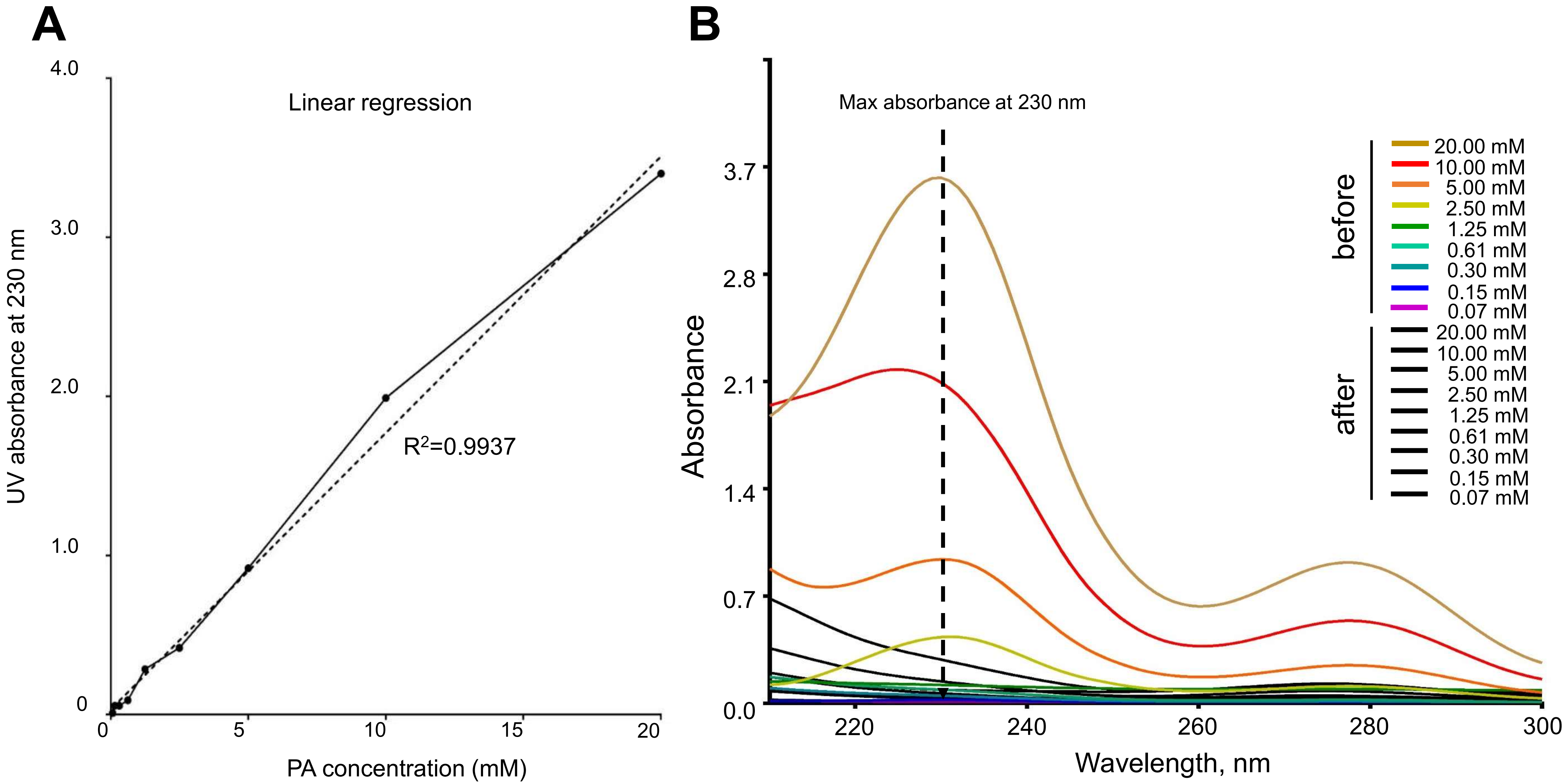

Fig. S4. The encapsulation capacity of proanthocyanidins (PA) into the ADC system. (A) UV absorbance intensity as a function of PA concentration in solution. (B) The UV–Vis spectra of PA solution before and after the loading into ADC system.

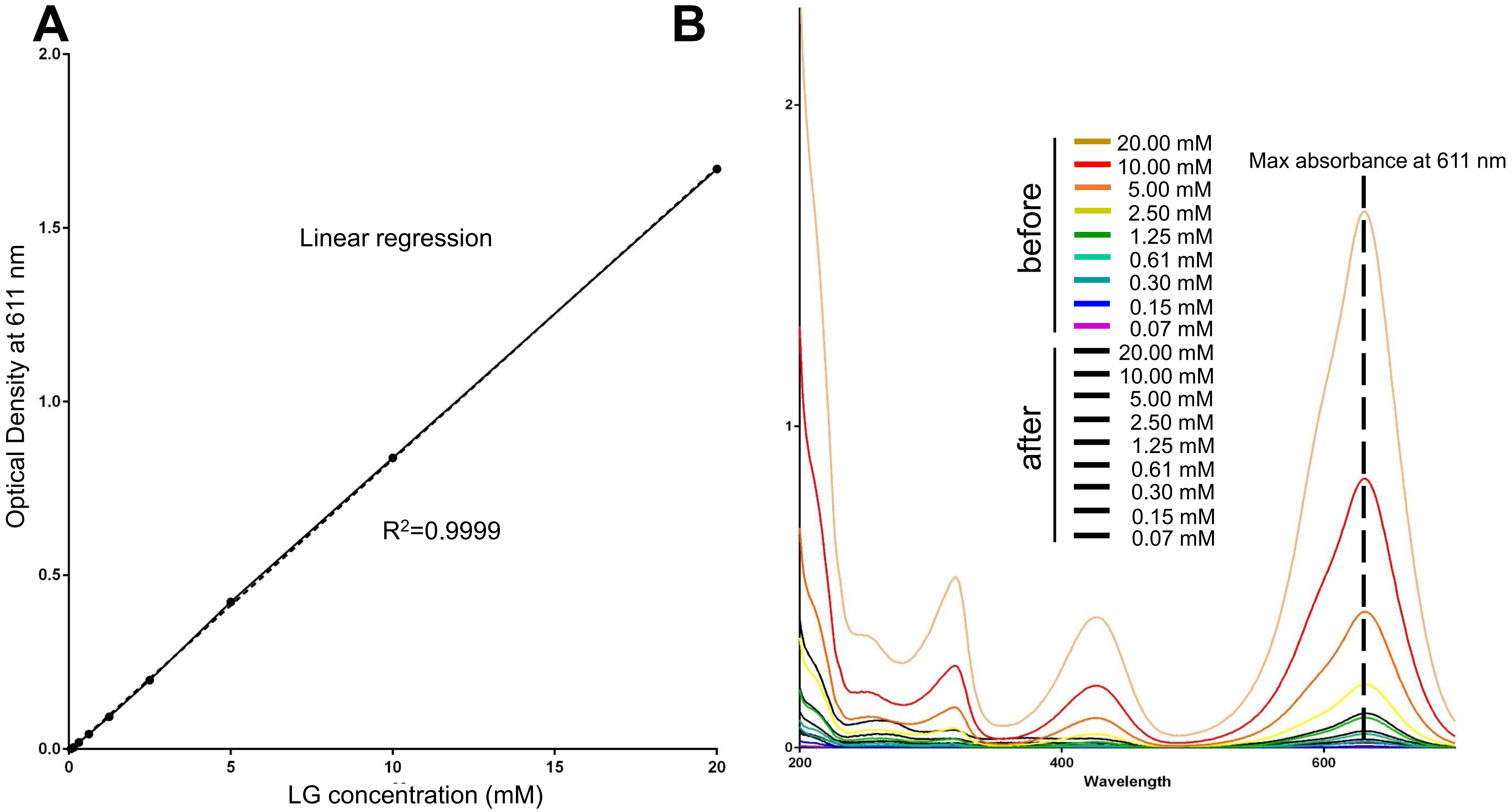

Fig. S5. The encapsulation capacity of light green SF (LG) into the ADC system. (A) UV absorbance intensity as a function of LG concentration in solution. (B) The UV–Vis spectra of LG solution before and after the loading into ADC system.

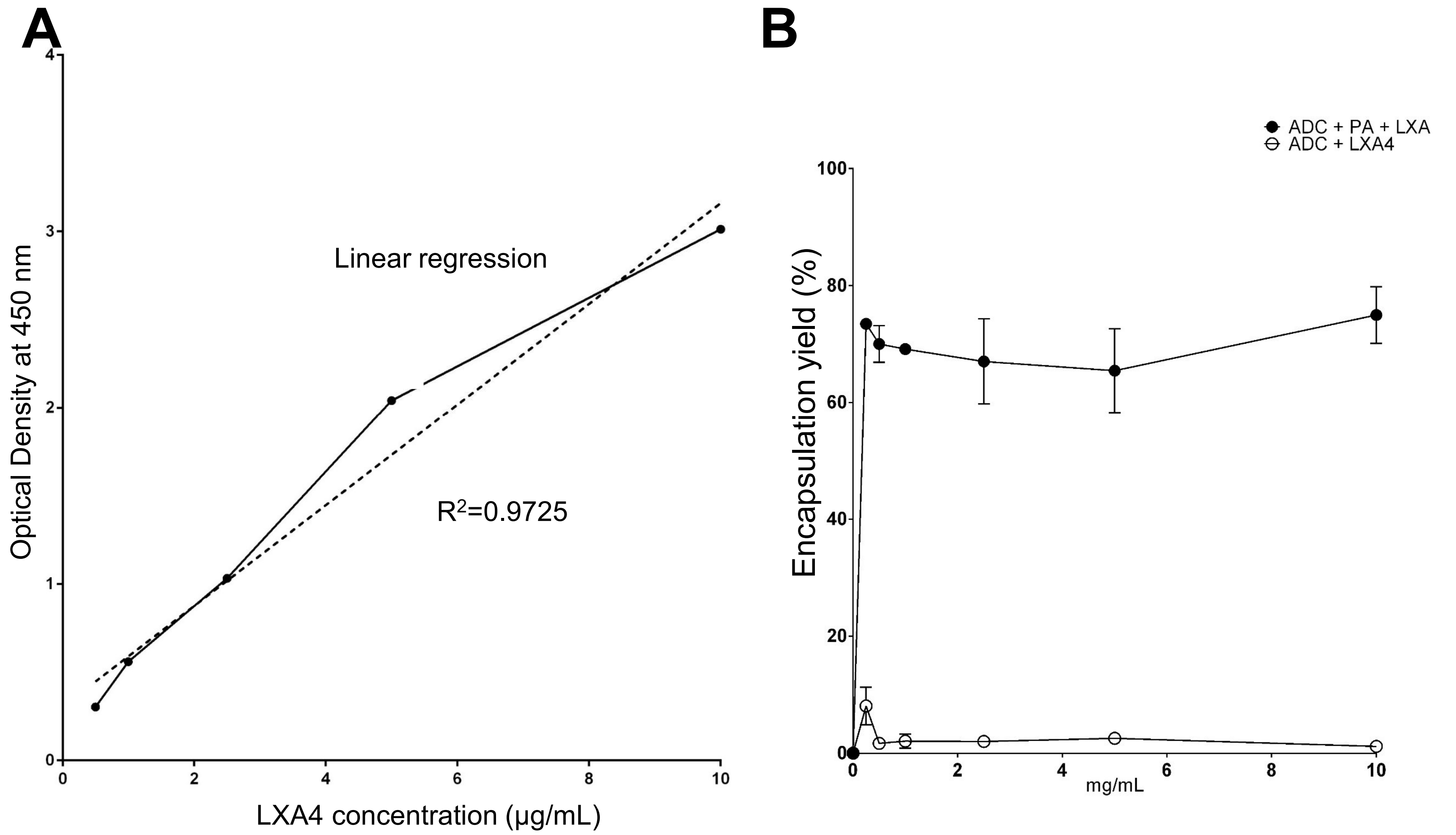

Fig. S6. The encapsulation capacity of lipid mediator lipoxin A4 (LXA4) into the ADC system. (A) UV absorbance intensity as a function of LXA4 concentration in solution. (B) The ELISA result of LXA4 solution before and after the loading into ADC system.

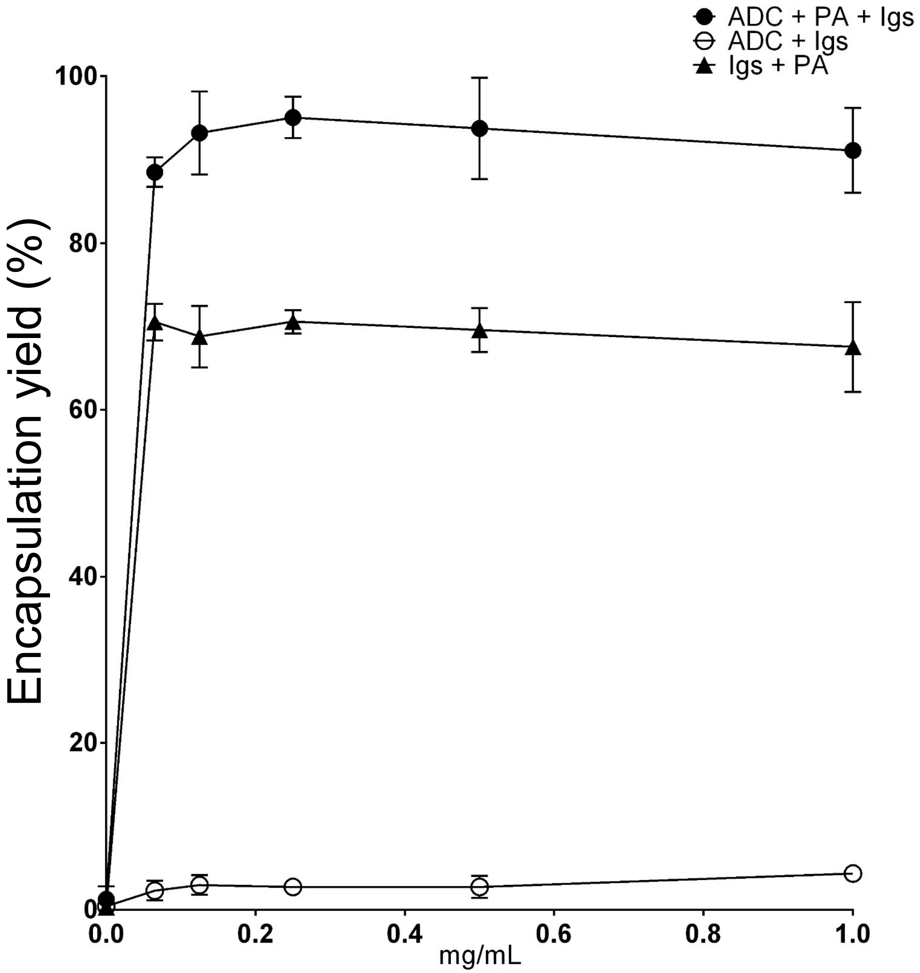

Fig. S7. The encapsulation capacity of fluorescent-labeled immunoglobulin (IgG) into the ADC system. The ELISA result of IgG solution before and after the loading into ADC system.

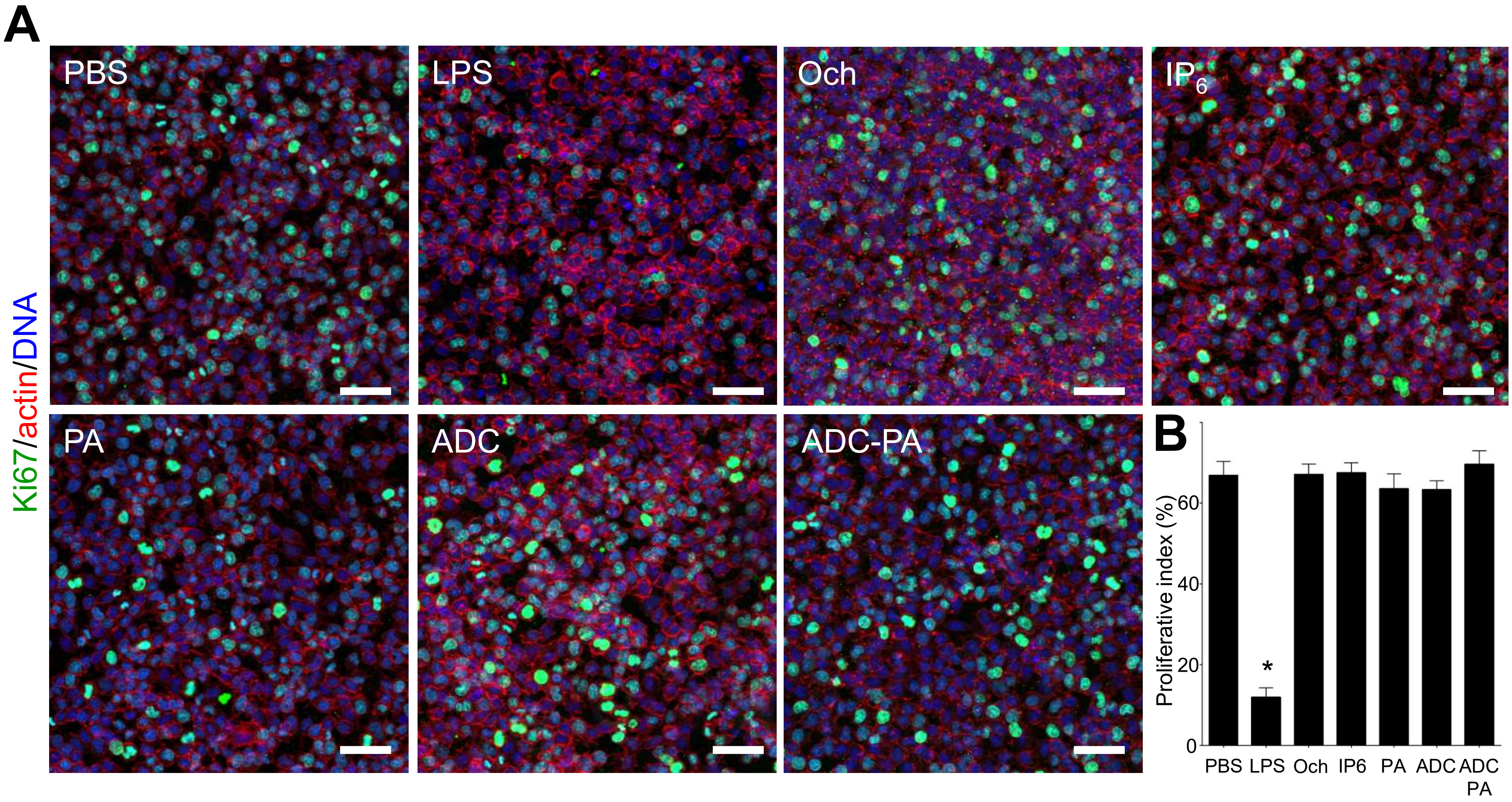

Fig. S8. Treatment with ADC or ADC-PA do not affect the proliferation in J774 macrophages. (A) Immunofluorescence staining of the proliferation marker Ki67 in each group: Actin (red), Ki67 (green), and DNA (blue). The results from three independent experiments and representative images are shown. Scale bars = 50 µm. (B) Statistical analysis of Ki-67-positive cells in each group. The results from three independent experiments are presented as the mean ± SD. *p < .05.

Supplementary Table. 1. Primers used in this study.

Genes	Forward (5'-3')	Reverse (5'-3')
Tnfα	CTGTAGCCCACGTCGTAGC	TTGAGATCCATGCCGTTG
Actin	GTAACAATGCCATGTTCAAT	CTCCATCGTGGGCCGCTCTAG
IL1β	TGTAATGAAAGACGGCACACC	TCTTCTTTGGGTATTGCTTGG
IL-6	TCCAGTTGCCTTCTTGGGAC	GTACTCCAGAAGACCAGAGG
Tgfβ	AAGTTGGCATGGTAGCCCTT	GCCCTGGATACCAACTATTGC
Arg-1	TTGGGTGGATGCTCACACTG	TTGCCCATGCAGATTCCC
Egr-2	CAGAGATGGGAGCGAAGCTA	TTGACCAGATGAACGGAGTG
inos	ACCAGTATAAGGCAAGCA	GCTTCTGGTCGA
Galectin-3	GGAGAGGGAATGATGTTGCCT	TCCTGCTTCGTGTTACACACA
Fizz-1	TCCCAGTGAATACTGATGAGA	CCACTCTGGATCTCCCAAGA
Ccl22	TGGAGTAGCTTCTTCACCCA	TCTGGACCTCAAAATCCTGC
C/EBPbeta	GGAGACGCAGCACAAGGT	AGCTGCTTGAACAAGTTCCG
Dectin-1	GACTTCAGCACTCAAGACATCC	TTGTGTCGCCAAAATGCTAGG
Sod1	GTGATTGGGATTGCGCAGTA	TGGTTTGAGGGTAGCAGATGAGT
Stat4	TTGAAGCAGAATTGTTGCCA	CTTTCCTATGGAAATCCGGC
Stat6	CTGGGGTGGTTTCCTCTTG	TGCCCGGTCTCACCTAACTA
5lox	CCCCGAGATATCCAGTTTGA	CCTGCCAGTGGTTCTTGACT
Ccl5	CCACTTCTTCTCTGGGTTGG	GTGCCCACGTCAAGGAGTAT
Cox2	CCCCCACAGTCAAAGACACT	CTCATCACCCCACTCAGGAT
Nlrp3	ATTACCCGCCCGAGAAAGG	CATGAGTGTGGCTAGATCCAAG
15lox	CAG GGA TCG GAG TAC ACG TT	GAT TGT GCC ATC CTT CCA GT
Sod3	CATGCAATCTGCAGGGTACAA	AGAACCAAGCCGGTGATCTG
Ccl17	ACCAGCTCACCAACTTCCTG	TGCTTCTGGGGACTTTTCTG
Irf-5	CAGGTTGGCCTTCCACTTG	ATGGGGACAACACCATCTTC
Pparg	AGTCCTCACAGCTGTTTGCCAAGC	GAGCGGGTGAAGACTCATGTCTGTC
Fibcd-1	GGCTCTGTGAACTTTTTCCGA	GAAGCTCGTATGCTGCTTGTG
Creb	ATCAGTTATCCAGTCTCCACAAGTCC	GTGATGGCAGGGGCTGAAGTC
Cd161	GTGCTGCTATCTCAGGAGACA	AACCCACATAGTTGCTCAGGG
Del-1	TGGCGGAATATGTACCGACCT	TCCCACTGTTACCTGAATCCA
Tlr2	CTCTTCAGCAAACGCTGTTCT	GGCGTCTCCCTCTATTGTATTG
cMyc	CGGACACACAACGTCTTGGAA	AGGATGTAGGCGGTGGCTTTT
Irf-4	GCCCAACAAGCTAGAAAG	TCTCTGAGGGTCTGGAAACT
Fpr2	CTTTTGGCTGGTTCCTGTGT	TTCCTAGCCAGGCTCACAGT
Stat1	CTGAATATTTCCCTCCTGGG	TCCCGTACAGATGTCCATGAT
Ym1	GGGCATACCTTTATCCTGAG	CCACTGAAGTCATCCATGTC
Mrc-1	GTGGATTGTCTTGTGGAGCA	TTGTGGTGAGCTGAAAGGTG
mTOR	TATTCCAACACCCCAGAAGC	CATATGCCAAAGCACTGCAC

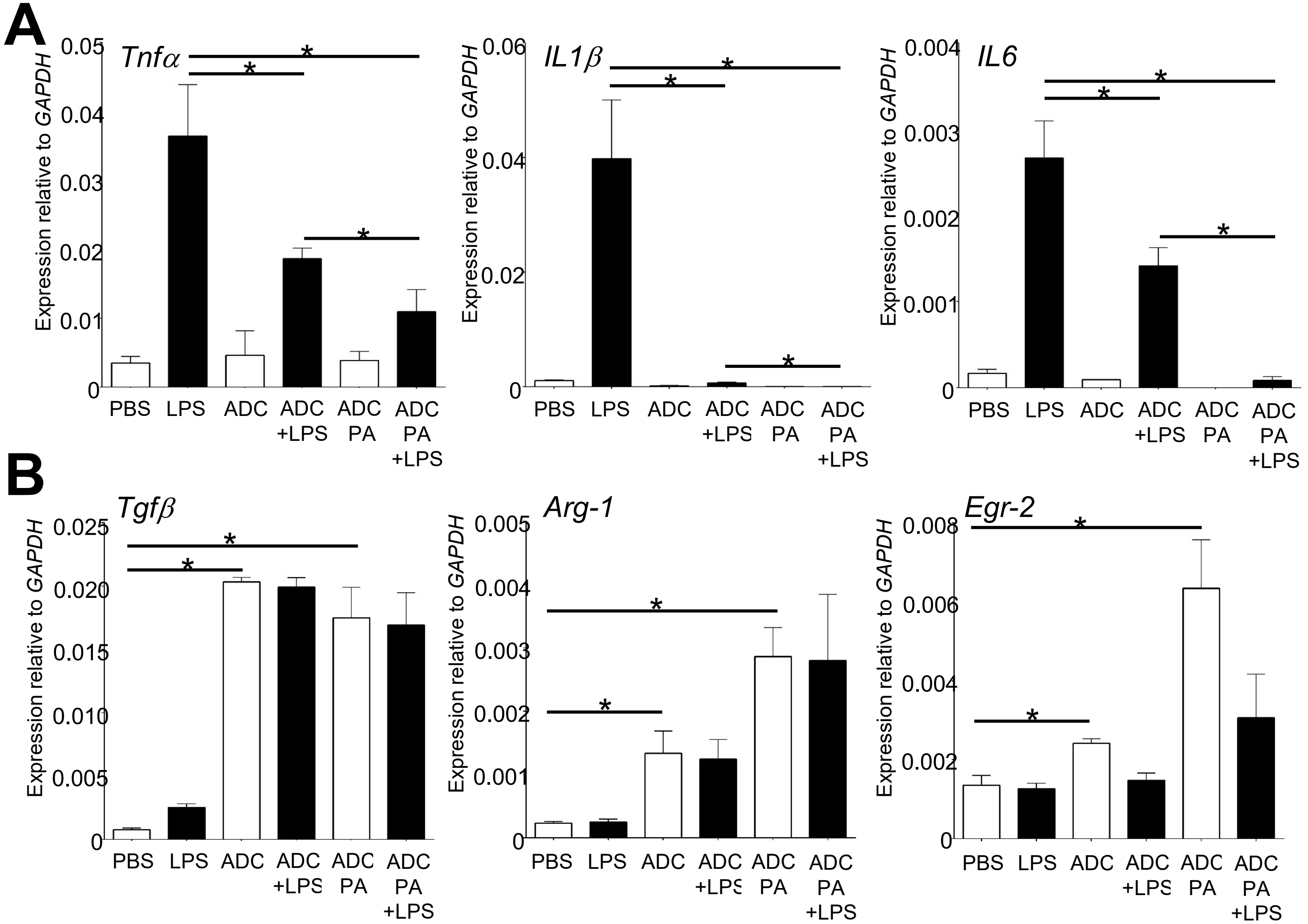

Fig. S9. Treatment with IP6 alters LPS-mediated gene expression in J774 macrophages. qPCR analysis of (A) pro-inflammatory cytokines and (B) anti-inflammatory cytokines in BMDMs treated with or without ADC or ADC-PA in the presence of LPS stimulation for 24 hr. The results from three independent experiments are presented as the mean ± SD. *p < .05.

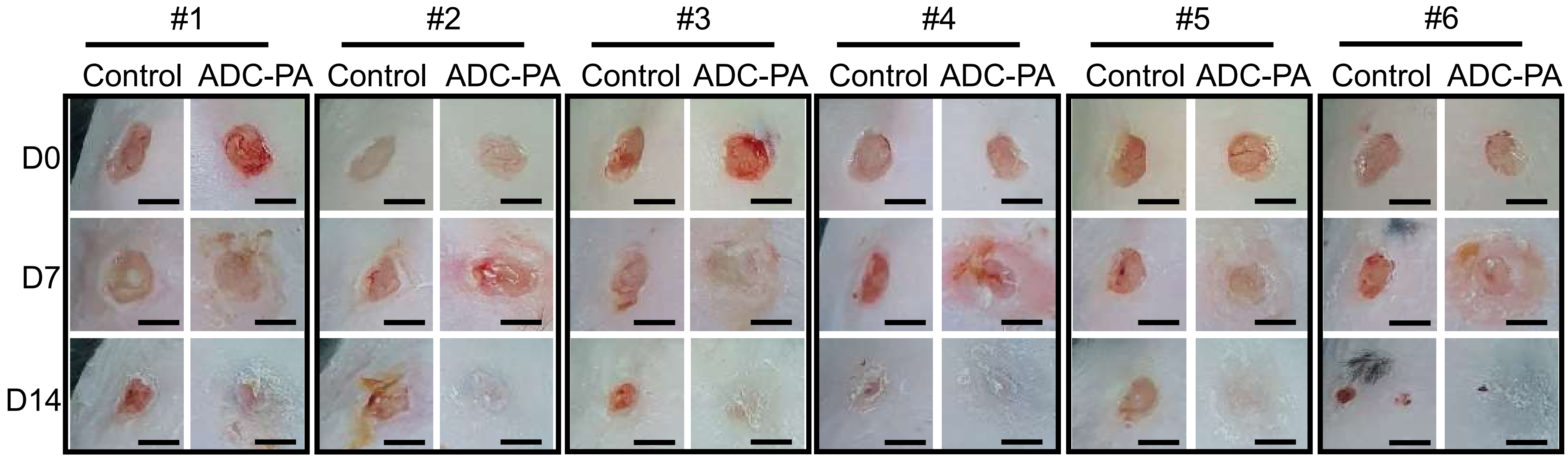

Fig. S10. Healing effects of AC-PA on full-thickness wounds in diabetic db/db mice. Images of wounds from each group over 14-day period post-wounding. Nexcare liquid bandage solution was used as control. Scale bar = 5 mm.

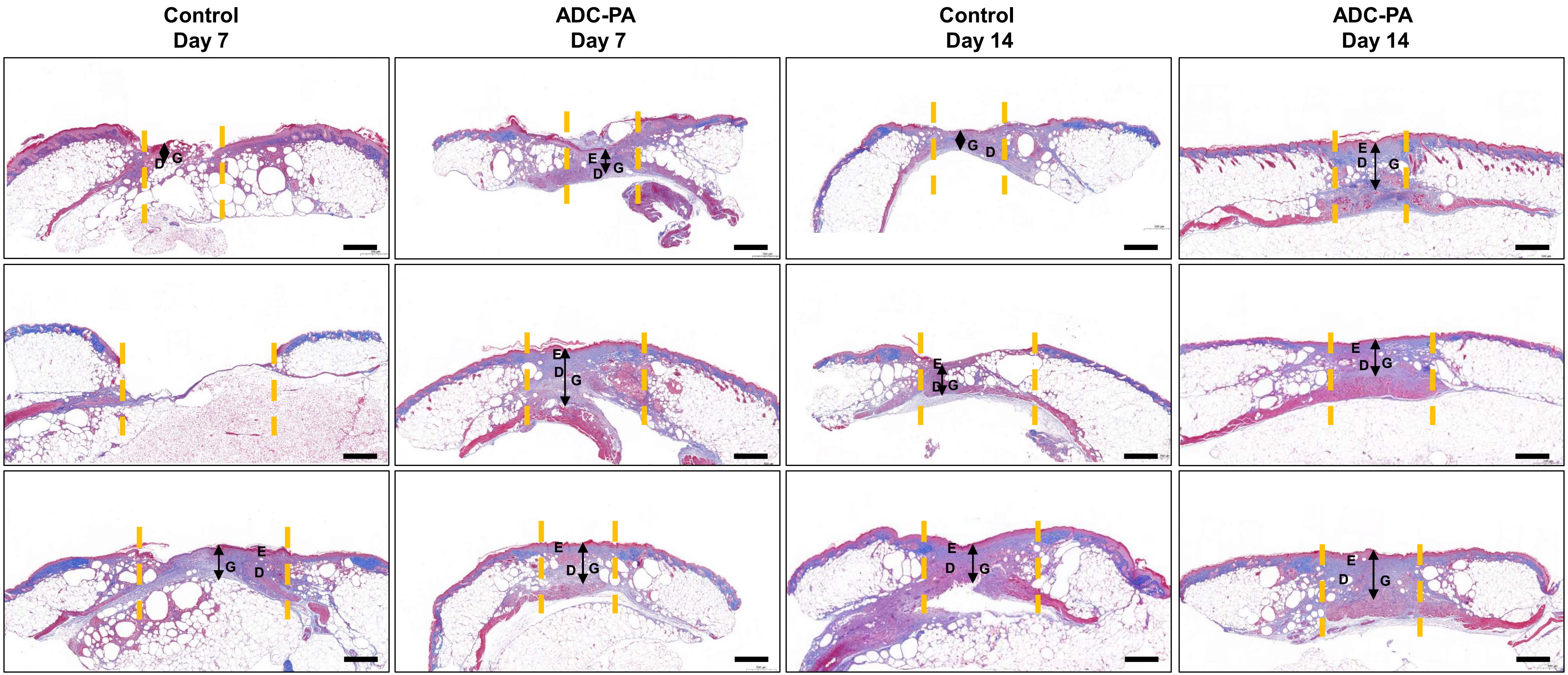

Fig. S11. Healing effects of AC-PA on full-thickness wounds in diabetic db/db mice. Masson's trichrome-stained skin tissue sections on day 7 and 14. Scale bar = 1 mm. Orange dash lines indicate the wound site. Yellow dash lines distinguish the border between epidermis and dermis. Abbreviations: E, epidermis; D, dermis; G, granulation area.

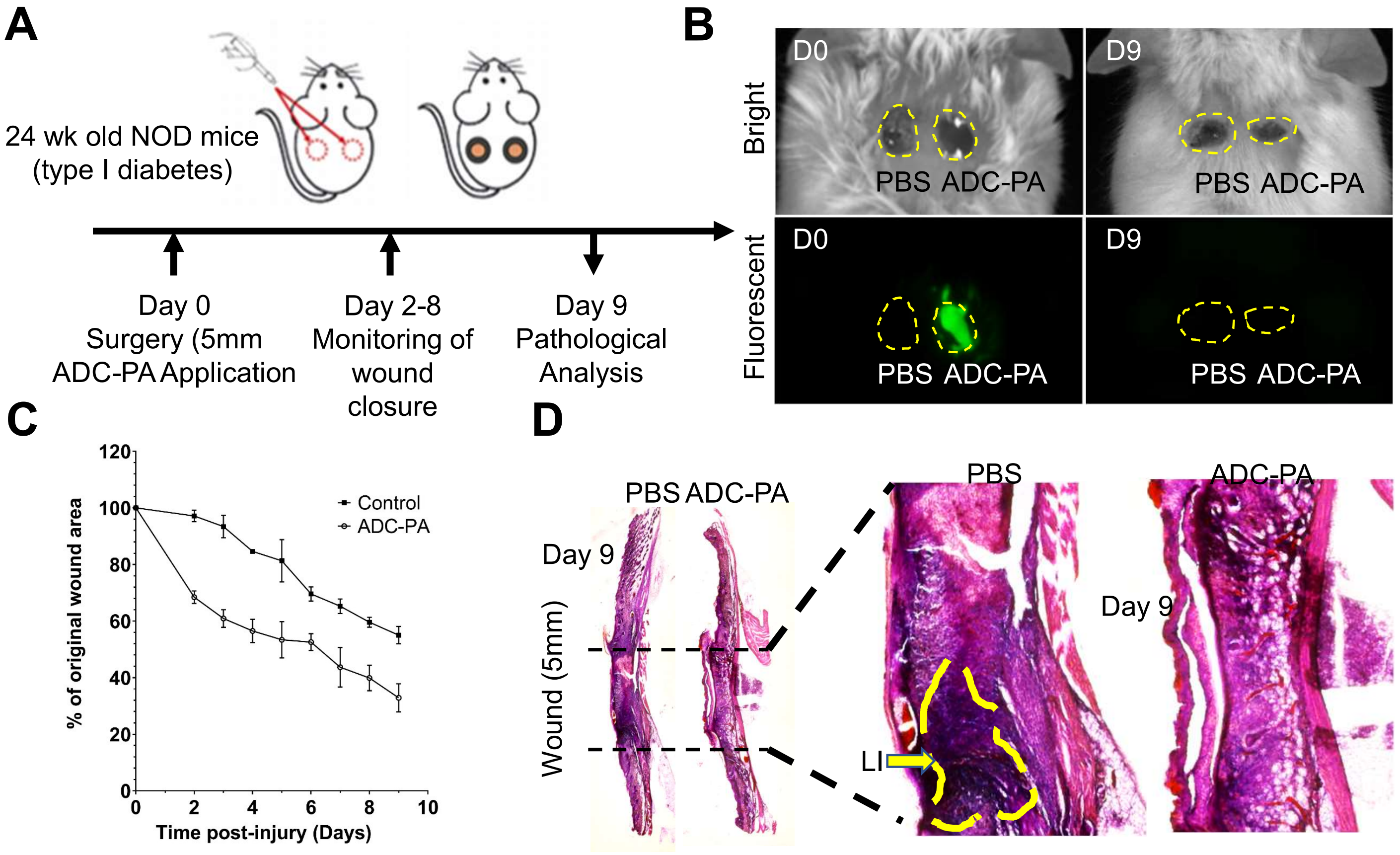

Fig. S12. ADC promotes skin wound healing in NOD mice. Twenty-four-week-old, male NOD mice were used as a model of wound healing. Briefly, full-skin-thickness excisional wounds (5 mm) were created on the dorsum of each mouse using a biopsy punch. (A) Wound conditions on days 0 and 9. ADC is depicted in green due to its autofluorescence. BF, bright-field. (B) Skin wound-closure rate in each group. Wound closure rate was measured daily and calculated based on wound area relative to the original size (D0). (C) Images of longitudinal sections of skin obtained from mice in each group and stained with hematoxylin and eosin (magnification, 4×). (D) Magnified images (20× magnification) of the areas outlined in black in (C). Yellow circle indicates the area of lymphocytic infiltration. *LI* lymphocytic infiltration.

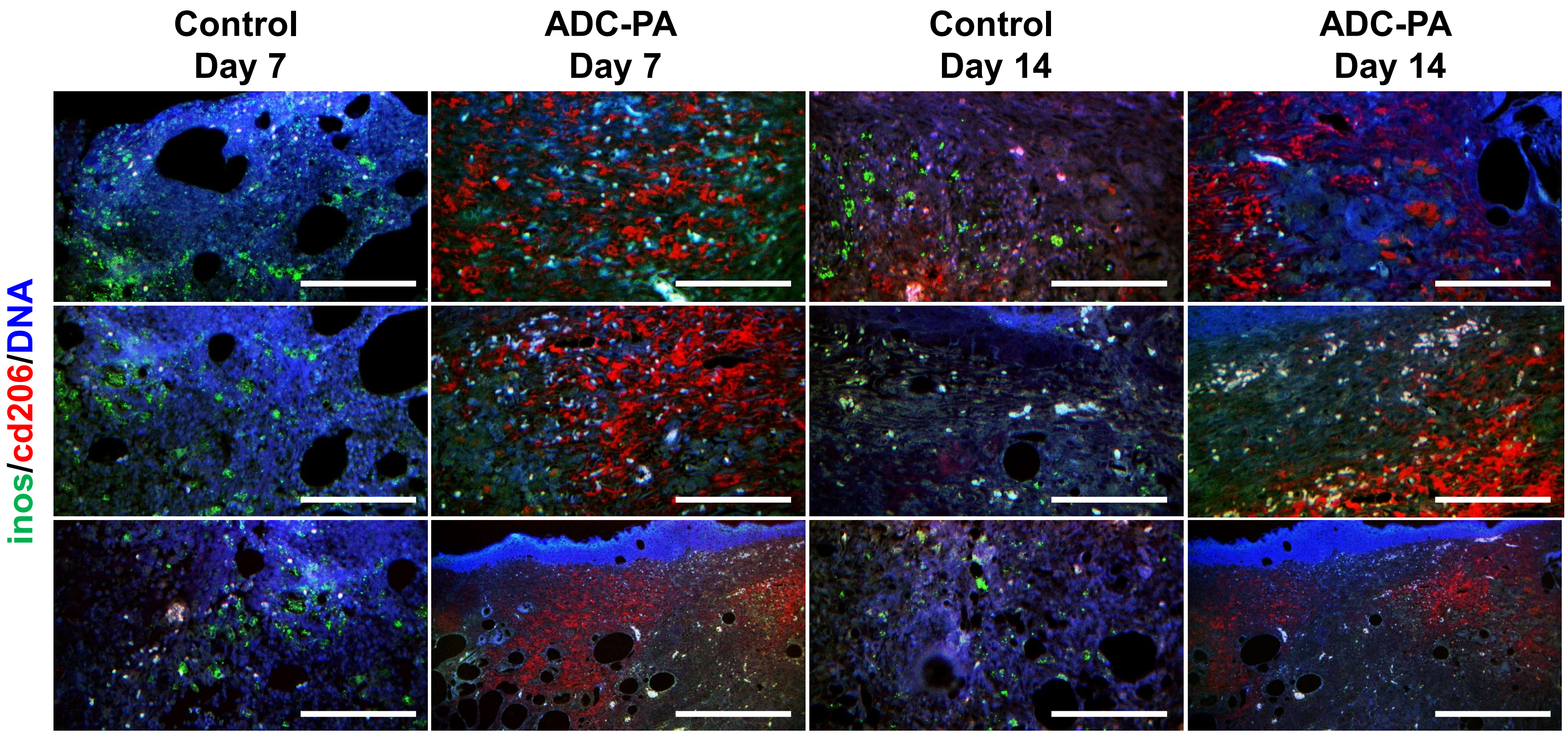

Fig. S13. Significant macrophage polarization from M1- to M2 subtype occurs in ADC-PA treated group. Fluorescence micrographs showed wounded skin immunostained with M1 marker iNOS (green) and M2 marker CD206 (red) at day 7 and 14. Scale bar = 200 μm. Abbreviations: iNOS, inducible nitric oxide synthase.
